# Adoptive Transfer of Immune Cells Into RAG2IL-2Rγ-Deficient Mice During *Litomosoides sigmodontis* Infection: A Novel Approach to Investigate Filarial-Specific Immune Responses

**DOI:** 10.3389/fimmu.2021.777860

**Published:** 2021-11-18

**Authors:** Anna Wiszniewsky, Laura E. Layland, Kathrin Arndts, Lisa M. Wadephul, Ruth S. E. Tamadaho, Dennis Borrero-Wolff, Valerine C. Chunda, Chi Anizette Kien, Achim Hoerauf, Samuel Wanji, Manuel Ritter

**Affiliations:** ^1^ Institute for Medical Microbiology, Immunology and Parasitology (IMMIP), University Hospital Bonn (UKB), Bonn, Germany; ^2^ German Centre for Infection Research (DZIF), Partner Site Bonn-Cologne, Bonn, Germany; ^3^ Parasite and Vector Biology Research Unit, Department of Microbiology and Parasitology, Faculty of Science, University of Buea, Buea, Cameroon; ^4^ Research Foundation for Tropical Diseases and the Environment (REFOTDE), Buea, Cameroon; ^5^ German-West African Centre for Global Health and Pandemic Prevention (G-WAC), Partner Site Bonn, Bonn, Germany

**Keywords:** Filariae, *Litomosoides sigmodontis*, CD4^+^ and CD8^+^ T cells, adoptive transfer, anti-filarial immunity, Th17 polarization

## Abstract

Despite long-term mass drug administration programmes, approximately 220 million people are still infected with filariae in endemic regions. Several research studies have characterized host immune responses but a major obstacle for research on human filariae has been the inability to obtain adult worms which in turn has hindered analysis on infection kinetics and immune signalling. Although the *Litomosoides sigmodontis* filarial mouse model is well-established, the complex immunological mechanisms associated with filarial control and disease progression remain unclear and translation to human infections is difficult, especially since human filarial infections in rodents are limited. To overcome these obstacles, we performed adoptive immune cell transfer experiments into RAG2IL-2Rγ-deficient C57BL/6 mice. These mice lack T, B and natural killer cells and are susceptible to infection with the human filaria *Loa loa*. In this study, we revealed a long-term release of *L. sigmodontis* offspring (microfilariae) in RAG2IL-2Rγ-deficient C57BL/6 mice, which contrasts to C57BL/6 mice which normally eliminate the parasites before patency. We further showed that CD4^+^ T cells isolated from acute *L. sigmodontis*-infected C57BL/6 donor mice or mice that already cleared the infection were able to eliminate the parasite and prevent inflammation at the site of infection. In addition, the clearance of the parasites was associated with Th17 polarization of the CD4^+^ T cells. Consequently, adoptive transfer of immune cell subsets into RAG2IL-2Rγ-deficient C57BL/6 mice will provide an optimal platform to decipher characteristics of distinct immune cells that are crucial for the immunity against rodent and human filarial infections and moreover, might be useful for preclinical research, especially about the efficacy of macrofilaricidal drugs.

## Introduction

Almost 4 decades ago, vector control and mass drug administration (MDA) programmes against lymphatic filariasis (LF) and onchocerciasis have been initiated to eliminate these diseases (1, 2). These programmes achieved a prevention of approximately 96 million new LF cases accompanied with a reduction from 120 to 68 million LF infections (3) and an interruption of *O. volvulus* transmission in Cuba, Ecuador, Mexico and Guatemala (4) and elimination in Mali and Senegal (5). However, worldwide elimination with the microfilaricidal and temporally embryostatic drug ivermectin that is currently used for MDA programmes will be impossible. Moreover, ivermectin cannot be applied in *Loa loa* endemic regions due to severe adverse events (6) and is insufficient against *Mansonella perstans* (7, 8), a filaria that is estimated to infect approximately 120 million people (9). Thus, research about anti-filarial immunity is urgently needed for the development of novel treatment strategies to fulfil the United Nations Sustainable Development Goals (SDG) 3.3. and end the epidemic of neglected tropical diseases (NTDs) by 2030.

Indeed, several studies have characterized host immunity during filarial infections and revealed that filarial infections dampen and modulate host immune responses *via* the induction of regulatory B and T cell subsets, Th2 cell populations and distinct patterns of cytokines, chemokines and immunoglobulins accompanied with the suppression of Th1 and pro-inflammatory cell subsets (10–18). This immunomodulation leads in most infected people to an asymptomatic clinical picture which is essential for long-term survival of the parasites (19, 20), but is also suggested to influence disease outcome of concomitant infections as well as vaccination efficacy (21–23). However, human studies are hindered especially due to the inability to obtain adult worms and the insufficient possibility to analyse infection kinetics and immune signalling. Although, *in vitro* culture models for human filariae have been developed (24–30), so far, it is impossible to obtain all life stages and mimic filarial life cycle, especially parasitic reproduction *in vitro*. Thus, several studies aimed to establish human filarial infections in rodent models (31–35), but so far, no complete life cycle could be established *in vivo*. Thus, the well-established murine model of filariasis *Litomosoides sigmodontis* is widely used to decipher filarial biology and filarial-driven immune modulation (36, 37). Interestingly, *L. sigmodontis* infection in BALB/c mice leads to patency including the release of microfilariae (MF) into the periphery, whereas C57BL/6 mice progressively eliminates adult worms before the onset of MF release (38–40). Studies using BALB/c mice revealed critical roles of CD4^+^ T cells (41), B1 cells (42), Th1 (43) and Th2 immune responses (44–47), eosinophils (48, 49) and neutrophils (50) for immunity against adult worms and MF. On the other hand, C57BL/6 mice are more characterized by a Th1/Th2 immune response (51) and research with this mouse strain mainly focused on earlier infection time-points. In brief, it has been shown that neutrophils (52, 53), Th17 signalling (54) and the secretion of IL-4 (44) influence worm development and the migration and survival of invading infective L3 larvae. However, information about the role of adaptive immune cells for anti-filarial immunity in C57BL/6 mice is missing. Interestingly, RAG2IL-2Rγ-deficient C57BL/6 mice, which lack T, B and natural killer cells (55), harbour high worm numbers and develop a 100% patency on day 72 p.i. (56), a time-point when wildtype C57BL/6 mice already cleared the infection. These findings suggest an important role of adaptive immune cells during *L. sigmodontis* infection in C57BL/6 mice. Thus, in this study we analysed the role of adaptive immunity in more detail and established adoptive T cell transfers into RAG2IL-2Rγ-deficient C57BL/6 mice to assess the influence of T cell subsets on parasitological, immunological and pathological aspects during a patent *L. sigmodontis* infection. Since RAG-deficient mice have been successfully infected with the human filaria *Loa loa* (34, 35) the implementation of the RAG2IL-2Rγ-deficient C57BL/6 mouse model in combination with adoptive transfer experiments might be a useful platform to investigate immunity during human filarial infections.

## Methods

### Mice

B6-Rag2^tm1Fwa^II2rg^tm1Wjl^ (RAG2IL-2Rγ^-/-^) mice were purchased from Taconic Biosciences Inc (Cologne, Germany). Wildtype C57BL/6J, C57BL/6-*Il4^tm1Nnt^*/J (IL-4^-/-^ C57BL/6) and B6.129P2-*Il10^tm1Cgn^*/J (IL-10^-/-^ C57BL/6) mice were purchased from Jackson Laboratories (Bar Harbor, USA). DEREG (Depletion of regulatory T cell) C57BL/6 mice were bred in house at the Institute for Medical Microbiology, Immunology and Parasitology (IMMIP), University Hospital Bonn (UKB), Bonn, Germany. Mice were kept under SPF conditions in accordance with German animal protection laws and EU guidelines 2010/63/E4 and had access to food and water ad libitum. All protocols and experiments were approved by the Landesamt für Natur, Umwelt und Verbraucherschutz Nordrhein-Westfalen, Recklinghausen, Germany (84.02.04.2017.A122).

### Experimental Setup of *Litomosoides sigmodontis* Infections and Adoptive T Cell Transfers

Natural infections with *L. sigmodontis* were performed through a bite of infected *Ornithonyssus bacoti mites* as previously described (57). Infected mice were analysed 28 or 72 days p.i. to isolate splenocytes for adoptive T cell transfer and to assess parasitological, immunological and pathological parameters. For the adoptive transfers, 1-3x10^6^ CD4^+^ or CD8^+^ T cells from naïve or 28- or 72-days *L. sigmodontis*-infected wildtype, IL-4^-/-^ or IL-10^-/-^C57BL/6 donor mice were injected intravenously (i.v.) into the tail vein of RAG2IL-2Rγ-deficient C57BL/6 mice one day prior to *L. sigmodontis* infection (**Supplementary Figure 1A**) or 28 or 49 days p.i. (**Supplementary Figure 1B**). In addition, adoptive transfer of T cells from DEREG (Depletion of regulatory T cell) C57BL/6 donor mice allows the depletion of regulatory T cells (Tregs) using diphtheria toxin (DTX) (58). Thus, 1µg DTX (Merck KGaA, Darmstadt, Germany) were intraperitoneal (i.p.) injected into adoptively transferred and *L. sigmodontis*-infected RAG2IL-2Rγ-deficient C57BL/6 mice on day 29 and 30 and day 49 and 50 p.i. (**Supplementary Figure 1C**), which corresponds to L4 moulting into adult worms and onset of MF, respectively (56).

### Isolation of CD4^+^ and CD8^+^ T Cells for Adoptive Transfer Experiments

To perform adoptive transfer experiment, spleens from naïve or *L. sigmodontis*-infected donor mice (day 28 or 72 p.i.) were isolated and mashed in sterile PBS (Thermo Fisher Scientific, Schwerte, Germany) using a sterile plunger (BD Bioscience, Heidelberg, Germany). Red blood cells in the cell suspension were lysed using ACT buffer (8.99g ammonium chloride + 2.06g Tris in 1l distilled water) for 6 minutes at room temperature (RT) with constant shaking. Afterwards, cells were passed through a gauze and washed with PBS (Thermo Fisher Scientific). To isolate T cells from the splenocyte suspension, magnetic cell separation (MACS) technique was applied using the mouse MHC class II MicroBeads Kit (Miltenyi Biotec B.V. & Co. KG, Bergisch Gladbach, Germany) according to manufacturer’s description. In brief, 1x10^7^ splenocytes were incubated with 10µl anti-mouse MHC class II beads for 15 minutes at 4°C. Upon washing of the bead-labelled cell suspension with auto MACS buffer (Miltenyi Biotec B.V. & Co. KG), cell suspension was applied on LS MACS columns (Miltenyi Biotec B.V. & Co. KG) and placed in the magnetic field of a MACS separator (Miltenyi Biotec B.V. & Co. KG). MHC class II negative cells were then flushed through the column using auto MACS buffer for further flow cytometry-based cell sorting. For flow cytometry-based CD4^+^ and CD8^+^ T cells sorting, Fc-receptors of the MHC class II negative cells were blocked with CD16/CD32 monoclonal antibody (Thermo Fisher Scientific) for 15 minutes and then labelled with APC- or FITC-conjugated anti-mouse CD4 (clone RM4-5; Biolegend, California, USA) or PerCP.Cy5.5-conjugated anti-mouse CD8 antibodies (clone 53-6.7; Biolegend). CD4^+^ and CD8^+^ T cells were then sorted using the BD FACS Aria III Cell Sorter (BD Bioscience) in the Flow Cytometry Core Facility at the University Hospital Bonn (UKB), Bonn, Germany to obtain a purity of CD4^+^ and CD8^+^ T cells of >96%.

### Assessment of Worm and Microfilarial Burden

*L. sigmodontis*-infected mice were sacrificed by inhaling Forene^®^ (Piramal Critical Care, West Drayton, UK) on day 30 or 72 p.i. Using sterile scissors and tweezers the mice were opened from the abdomen to the sternum without damaging the diaphragm. Then, a small orifice was made in the middle point of the upper border of the diaphragm and by using sterile plastic Pasteur pipettes (Ratiolab, Dreieich, Germany) filled with sterile PBS (Thermo Fisher Scientific), the thoracic cavity (TC) was washed several times. The collected fluid was passed through gauze to collect the *L. sigmodontis* worms and were immediately analysed for gender, developmental stage and length. For the later, we determined individual worm length and average of worm lengths per mouse (worm length per individual mouse). In addition, approximately 5 female worms/mouse were snap frozen in liquid nitrogen and then stored at -20°C for analysis of embryonic stages. To analyse MF counts, 25µl TC fluid were transferred into 300µl Hinkelmann solution (Merck KGaA, Darmstadt, Germany) and incubated for 10 minutes at RT. Upon centrifugation for 5 minutes at 1300rpm using the Eppendorf 5415 R centrifuge (Eppendorf AG, Hamburg, Germany), the sediment was microscopically assessed for the present of MF using the Leica DM IL microscope (Leica Microsystems GmbH, Wetzlar, Germany). In addition, 25µl peripheral blood was also taken from the cheek vein of *L. sigmodontis*-infected mice on day 50, 56, 63 and 72 p.i. to assess MF counts in the periphery using the above explained Hinkelmann staining protocol.

### Embryogram of Female Adult Worms

To analyse the embryonic stages, single female worms were squeezed in 80μl PBS (Thermo Fisher Scientific) and incubated with 20µl Hinkelmann solution (Merck KGaA). Then, 10μl of solution was microscopically analysed using the Leica DM IL microscope (Leica Microsystems GmbH) to assess presence and numbers of eggs, morulae, pretzel and stretched MF (**Supplementary Figure 2**).

### Assessment of Immune Cell Infiltration Between Lung and Diaphragm

To assess immune cell infiltration and resulting pathology between the lung and diaphragm, close to the site of infection, the thoracic cavity (46), organs from *L. sigmodontis*-infected RAG2IL-2Rγ-deficient C57BL/6 mice on day 72 p.i, were embedded in paraffin to prepare 3µm sections using the Rotary 3005E semi- electronic rotary microtome (PFM Medical, Cologne, Germany). Sections were then stained with hematoxylin and eosin (HE) as previously described (59) and microscopically examined to assess the inflammation score on a scale from 0-4 (0 = no cell infiltration; 1 = one to two cell layers; 2 = three to four cell layers; 3 = five to six cell layers; 4 = more than six cell layers; infiltrated cells widespread over the whole tissue) by the degree of visual thickness and infiltration of immune cells towards the outside of the lung and diaphragm (46).

### Assessment of Immune Cell Composition in the Thoracic Cavity (TC)

To determine the immune cell composition in the TC, fluid was centrifuged at 1200 rpm for 5 minutes using the Multifuge 4KR (Heraeus, Hanau, Germany). Then supernatant was discarded and cell pellet resuspended in 1ml PBS (Thermo Fisher Scientific) to determine cell counts using trypan blue. 5x10^4^ TC cells were then pulse centrifuged on glass slides using the Cytospin^®^ technique as previously described (46). Dried cytospin slides were stained using the Diff-Quik staining set (Medion Diagnostics, Miami, USA) according to the manufacturer’s instructions. To determine immune cell composition at least 100 cells were microscopically differentiated into lymphocytes, macrophages, neutrophils and eosinophils using the Leica DM IL microscope (Leica Microsystems GmbH).

### Flow Cytometry

For detailed analysis of the adoptively transferred CD4^+^ T cells, red blood cells from TC were eliminated by using ACT buffer for 6 minutes at RT with constant shaking. Upon washing in PBS (Thermo Fisher Scientific), remaining cells were fixed and permeabilized using eBioscience™ fixation/permeabilization concentrate and permeabilization buffer (Thermo Fisher Scientific) according to the manufacture’s description. After block of the Fc receptors, TC cells were stained with combinations of fluorophore (APC, FITC, PE, PE-Cy7, PerCp.Cy5.5)-conjugated anti-mouse CD4 (clone RM4-5), IFN-γ (clone XMG1.2), IL-4 (clone 11B11), IL-10 (clone Jes5-16E3) and IL-22 (clone Poly5164) monoclonal antibodies from Biolegend and IL-5 (clone TRFK5) and IL-17A (clone eBio17B7) monoclonal antibodies from Thermo Fisher Scientific. Samples were acquired using the FACS Canto I (BD Bioscience) or the CytoFlex S cytometer (Beckman Coulter, Krefeld, Germany) and compensation was performed using BD™ CompBead particles (BD Biosciences) or the VersaComp antibody capture kit (Beckman Coulter). In addition, fluorescence minus one (FMO) controls were acquired to discriminate populations. Finally, antibody expression levels were analysed using the FlowJo v10 software (FlowJo, Portland, USA). **Supplementary Figure 3** shows the applied gating strategy.

### Analysis of Cytokine and Chemokine Levels in TC

On analysis days, the first 500µl of the TC fluid was collected during the flushing of *L. sigmodontis* worms. To measure cytokines and chemokines the Cytokine & Chemokine 36-Plex Mouse ProcartaPlex™ Panel 1A Luminex kit (Thermo Fisher Scientific) was applied according to manufacturer’s descriptions. The limits (upper limit of quantification/lower limit of quantification) of the cytokines and chemokines in pg/ml were as follows: CCL2 (9,850/9.62), CCL3 (613/0.60), CCL4 (863/0.84), CCL5 (2,600/2.54), CCL7 (1,000/0.24), CCL11 (738/0.72), CXCL1 (2,550/2.49), CXCL2 (1,038/1.01), CXCL5 (27,600/6.74), CXCL10 (750/0.73), G-CSF (1,375/1.34), GM-CSF (3,275/3.20), IFN-α (3,600/3.52), IFN-γ (8,950/2.19), IL-1α (13,400/3.27), IL-1β (5,900/1.44), IL-2 (1,913/1.87), IL-3 (1,550/0.38), IL-4 (5,600/1.37), IL-5 (8,950/2.19), IL-6 (22,600/5.62), IL-9 (21,850/21), IL-10 (8,350/2.04), IL-12p70 (600/2.34), IL-13 (2,925/2.86), IL-15 (8,150/1.99), IL-17A (2,500/2.44), IL-18 (145,700/36), IL-22 (53,200/13), IL-23 (47,600/12), IL-27 (10,800/2.64), IL-28 (130,400/32), IL-31 (10,325/10), LIF (6,800/1.66), M-CSF (750/0.18) and TNF-α (3,750/3.66). Samples were acquired using the MAGPIX Luminex system (Luminex Cooperation, Austin, USA) and analysed with ProcartaPlex Analyst software 1.0 (Thermo Fisher Scientific).

### Statistical Analysis

Statistical analyses were performed using the PRISM 7 programme (GraphPad Software, Inc., La Jolla, USA). Before testing for statistical significances between the groups, we performed a D’Agostino-Person omnibus normality test to test the distribution of the values. Since variables were non-parametrical distributed, Kruskal-Wallis-tests were performed to compare more than two groups. If a Kruskal-Wallis-test was significant a Dunn’s multiple comparison test was performed for a further comparison of the groups. P-values of 0.05 or less were considered significant.

## Results

### Investigation of *L. sigmodontis* Immunity Using Adoptive Immune Cell Transfers Into RAG2IL2Rγ-Deficient Mice

**Figure 1:**
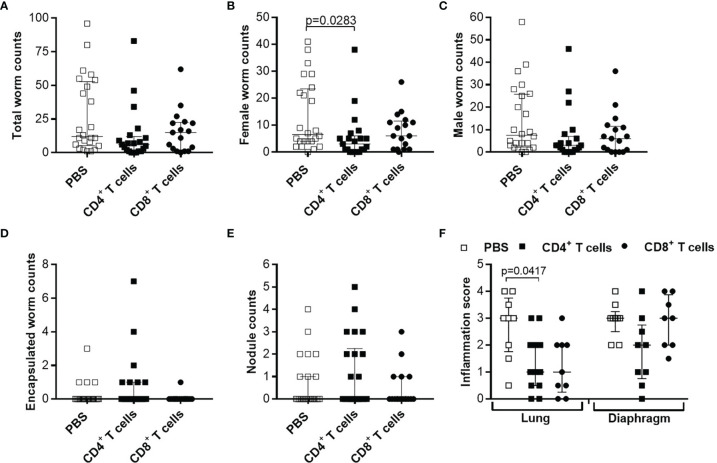
Adoptive transfer of naïve T cells do not alter parasite burden and thoracic cavity pathology in *L. sigmodontis*-infected RAG2IL-2Rγ-deficient C57BL/6 mice. CD4^+^ or CD8^+^ T cells from naïve C57BL/6 donor mice were injected intravenously (i.v.) into the tail vein of RAG2IL-2Rγ-deficient C57BL/6 mice one day prior to *L. sigmodontis* infection. On day 72 p.i. mice were analysed to assess **(A)** total, **(B)** female, **(C)** male and **(D)** encapsulated worm counts as well as **(E)** nodule counts in the thoracic cavity. In addition, H&E-stained lung and diaphragm sections were microscopically analysed to assess the **(F)** inflammation score. Graphs show dot blots with median and interquartile ranges from individual mice from **(A–E)** four independent experiments including (no adoptive transfer (PBS; n=25) and adoptive transfer of CD4^+^ (n=21) and CD8^+^ T cells (n=17) and **(F)** two independent experiments including PBS (n=9) and adoptive transfer of CD4^+^ (n=12) and CD8^+^ T cells (n=9) into RAG2IL-2Rγ-deficient C57BL/6 mice. Significant differences between the groups were determined by Kruskal-Wallis-test followed by a Dunn’s multiple comparison test.

Our previous study showed that *L. sigmodontis* infections in RAG2IL-2Rγ-deficient C57BL/6 mice led to high worm burden and that all infected mice release MF at the site of infection and periphery 72 days p.i. (56), a time-point when wildtype C57BL/6 mice have already cleared the infection. Moreover, MF secretion in the periphery peaks between day 70-120 p.i. and MF persist for up to 300 days p.i. in the periphery (**Supplementary Figure 4**). These results show that *L. sigmodontis* worms can survive and produce MF in RAG2IL-2Rγ-deficient C57BL/6 mice for a longer period of time in contrast to other mouse systems (38) and are comparable to infections in *Mastomoys coucha* (60). Since RAG2IL-2Rγ-deficient C57BL/6 mice lack T, B and natural killer cells (55), we suggested that adoptive cell transfer experiments might reveal crucial immune cell subsets and immune mechanisms that are important for immunity against filariae. Previous research revealed a crucial role for T cells for host immunity in BALB/c mice (41) but information is lacking for C57BL/6 mice. Therefore, we adoptively transferred CD4^+^ and CD8^+^ T cells from naïve C57BL/6 donor mice into RAG2IL-2Rγ-deficient C57BL/6 mice one day prior *L. sigmodontis* infection and analysed worm burden at 72 days p.i. (**Supplementary Figure 1A**), a time-point when RAG2IL-2Rγ-deficient C57BL/6 mice have MF in the periphery (**Supplementary Figure 4**). Despite a slight reduction in female worm numbers in mice that received naïve CD4^+^ T cells, parasite burden as well as worms that are surrounded by immune cells (encapsulated worms) and finally become granulomatous nodules remain comparable between the groups (**Figures 1A–E**). In addition, infiltration of immune cells and inflammation between lung and diaphragm, close to the thoracic cavity (TC), the site of infection, was only slightly reduced especially around lung tissue upon adoptive transfer of naïve CD4^+^ T cells (**Figure 1F**), suggesting that naïve CD4^+^ or CD8^+^ T cell transfer is not sufficient to significantly reduce worm burden and inflammation in the TC.

### Reduced Worm Length and MF Secretion Accompanied With Impaired Embryogenesis in RAG2IL2Rγ-Deficient Mice Upon Adoptive Transfer of CD4^+^ T Cells From Naïve C57BL/6 Donor Mice

**Figure 2:**
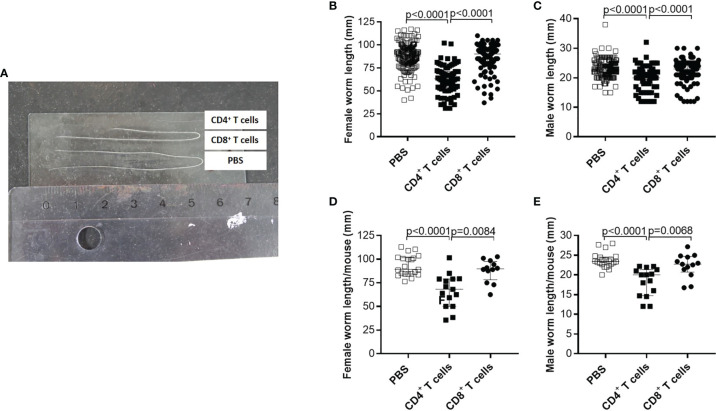
Adoptive transfer of naïve CD4^+^ T cells impair worm growth. On day 72 p.i., RAG2IL-2Rγ-deficient C57BL/6 mice that were adoptively transferred with CD4^+^ or CD8^+^ T cell from naïve C57BL/6 donor mice were analysed for **(A)** worm length to assess individual **(B)** female and **(C)** male worm length and **(D)** female and **(E)** male worm length per mouse. **(B–E)** Graphs show dot blots with median and interquartile ranges and are pooled from four independent experiments including no adoptive transfer (PBS; n=25) and adoptive transfer of CD4^+^ (n=21) and CD8^+^ T cell (n=17) into RAG2IL-2Rγ-deficient C57BL/6 mice. Significant differences between the groups were determined by Kruskal-Wallis-test followed by a Dunn’s multiple comparison test.

**Figure 3:**
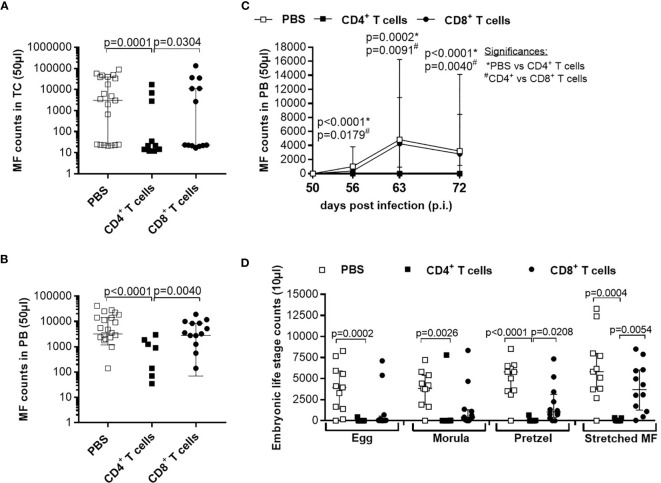
Adoptive transfer of naïve CD4^+^ T cells impair embryogenic stage development accompanied with reduce microfilariae release. Microfilaria (MF) counts from RAG2IL-2Rγ-deficient C57BL/6 mice that were adoptively transferred with CD4^+^ or CD8^+^ T cell from naïve C57BL/6 donor mice were determined in the **(A)** thoracic cavity (TC) and **(B)** peripheral blood (PB) on day 72 p.i. and **(C)** during the course of infection in PB upon 50 days p.i. Moreover, female adult worms were analysed to determine **(D)** embryonic stages. Graphs show dot blots with median and interquartile ranges. **(A–C)** Results are pooled from four independent experiments including no adoptive transfer (PBS; n=25) and adoptive transfer of CD4^+^ (n=21) and CD8^+^ T cell (n=17) into RAG2IL-2Rγ-deficient C57BL/6 mice. **(D)** Results are from one experiment including n=9, n=6 and n=11 individual worms from no adoptive transferred (PBS; n=5) and CD4^+^ (n=6) and CD8^+^ T cell (n=6) adoptive transferred RAG2IL-2Rγ-deficient C57BL/6 mice, respectively. Significant differences between the groups were determined by Kruskal-Wallis-test followed by a Dunn’s multiple comparison test.

Although parasite burden and inflammation score at the site of infection were not significantly altered upon adoptive T cell transfer from naïve donor mice, we further analysed the morphology of the worms and indicate that adoptive transfer of naïve CD4^+^ but not CD8^+^ T cells led to significantly shorter worms (**Figure 2A**). Detailed analysis of individual worm lengths (**Figures 2B, C**) and the average worm length per individual mouse (**Figures 2D, E**) showed a significant length reduction only upon CD4^+^ T cell transfer. Furthermore, we determined that adoptive transfer of naive CD4^+^ but not CD8^+^ T cells significantly reduced MF count at the site of infection (**Figure 3A**) and in the periphery (**Figure 3B**) on day 72 p.i. The significant reduction of MF on the analysis day was reflected during the course of infection and already at the onset of MF secretion in the periphery from day 56 p.i. (**Figure 3C**). Since MF numbers in the TC and periphery were significantly reduced upon CD4^+^ T cell transfer, we analysed female worms to assess embryonic stages and revealed also significantly reduced numbers of eggs, morulae, pretzels and stretched microfilariae (**Supplementary Figure 2**) in female worms from RAG2IL2Rγ-deficient mice that were adoptively transferred with naïve CD4^+^ T cells (**Figure 3D**). Collectively, these results indicate that despite the fact that adult worm numbers remain comparable, adoptive CD4^+^ T cell, but not CD8^+^ T cell transfer from naïve C57BL/6 donor mice induce immunity against *L. sigmodontis* leading to reduced worm length and MF numbers accompanied with impaired embryogenesis.

### Role of IL-4, IL-10 and Regulatory T Cells During Adoptive Transfer of CD4^+^ T Cells From Naïve C57BL/6 Donor Mice

As shown in **Figures 2**, **3**, adoptive transfer of CD4^+^ T cells from naïve C57BL/6 donor mice impairs the growth and MF production of adult worms. Previous studies showed that IL-4 (44) and IL-10 (61) influence *L. sigmodontis* worm development in C57BL/6 mice. Thus, we adoptively transferred CD4^+^ T cells from IL-4- and IL-10 -deficient C57BL/6 donor mice to assess the role of these cytokines (**Supplementary Figure 1A**). Interestingly, despite significantly smaller female (**Supplementary Figure 5F**) or male worms (**Supplementary Figure 6G**) in RAG2IL-2Rγ-deficient C57BL/6 mice that were adoptively transferred with IL-4^-/-^ or IL-10^-/-^ CD4^+^ T cells respectively, comparable worm and MF numbers as well as worm lengths were determined between adoptive transfer of naïve wildtype and IL-4^-/-^ (**Supplementary Figure 5**) or IL-10^-/-^ CD4^+^ T cells (**Supplementary Figure 6**). In addition, IL-10 is an important cytokine of regulatory T cells (Tregs) that has been shown to play a crucial role during filarial-specific immune responses (62). To investigate the role of Tregs, we adoptively transferred naïve CD4^+^ T cell from DEREG C57BL/6 donor mice and specifically depleted Tregs on day 29 and 30 (L4 moulting into adult worms) and day 49 and 50 (onset of MF) *via* the injection of diphtheria toxin (**Supplementary Figure 1C**). However, Treg depletion did not alter the results, since comparable worm and MF burden and also length of adult worms were revealed between CD4^+^ and Treg-depleted CD4^+^ T cell transferred mice (**Supplementary Figure 7**). In summary, these results suggest that the ability of naïve CD4^+^ T cells to reduce worm length and MF numbers is independent of IL-4 and IL-10 signalling or the involvement of Tregs.

### Adoptive Transfer of CD4^+^ T Cells From *L. sigmodontis*-Infected C57BL/6 Donor Mice Effectively Clears the Infection and Reduces Inflammation at the Site of Infection

**Figure 4:**
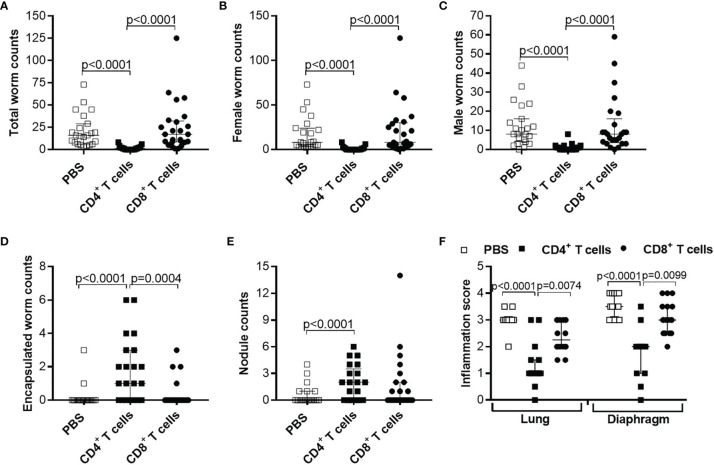
Reduced worm counts and thoracic cavity pathology in *L. sigmodontis*-infected RAG2IL-2Rγ^-/-^ mice upon adoptive transfer of CD4^+^ T cell from *L. sigmodontis*-infected C57BL/6 donor mice. CD4^+^ or CD8^+^ T cells from C57BL/6 donor mice that were infected with *L. sigmodontis* for 28 days were injected intravenously (i.v.) into the tail vein of RAG2IL-2Rγ-deficient C57BL/6 mice one day prior to *L. sigmodontis* infection. On day 72 p.i. mice were analysed to assess **(A)** total, **(B)** female, **(C)** male and **(D)** encapsulated worm counts as well as **(E)** nodule counts in the thoracic cavity. In addition, H&E-stained lung and diaphragm sections were microscopically analysed to assess the **(F)** inflammation score. Graphs show dot blots with median and interquartile ranges from individual mice from **(A–E)** three independent experiments including no adoptive transfer (PBS; n=23) and adoptive transfer of CD4^+^ (n=21) and CD8^+^ T cells (n=25) and **(F)** two independent experiments including PBS (n=10) and adoptive transfer of CD4^+^ (n=15) and CD8^+^ T cells (n=16) into RAG2IL-2Rγ-deficient C57BL/6 mice. Significant differences between the groups were determined by Kruskal-Wallis-test followed by a Dunn’s multiple comparison test.

**Figure 5:**
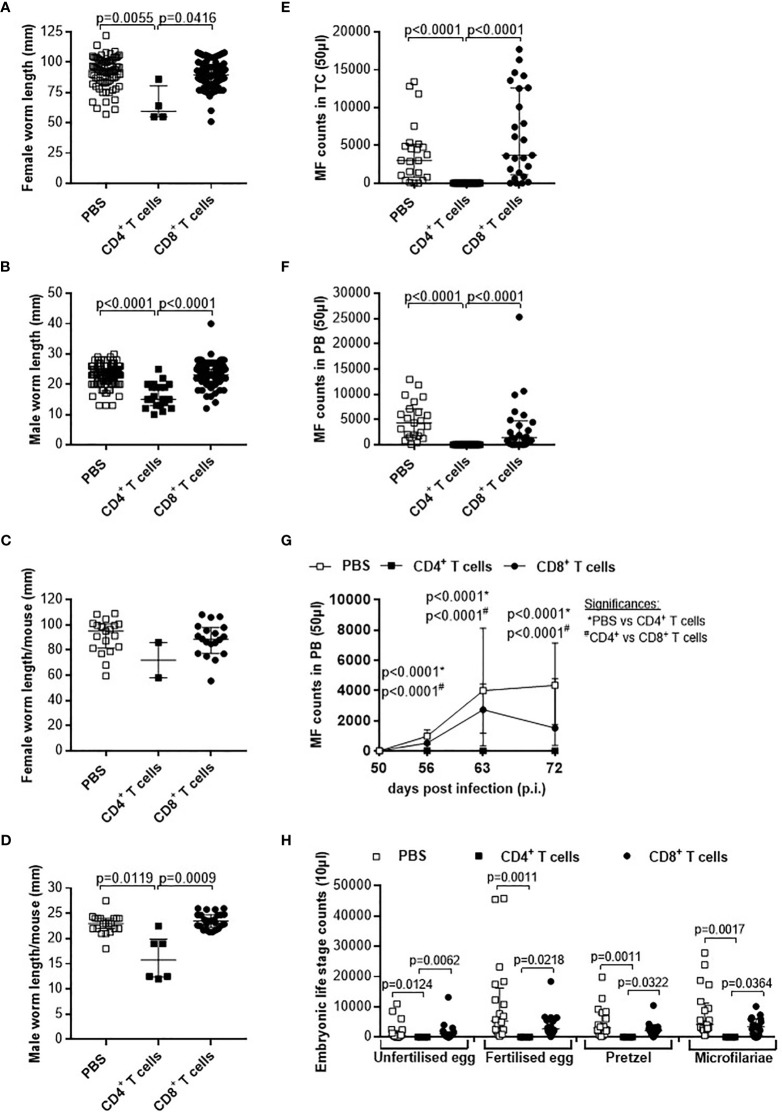
Adoptive transfer of CD4^+^ T cells from *L. sigmodontis*-infected C57BL/6 mice impair worm growth and blocks microfilaria release and development of embryogenic stages. On day 72 p.i., RAG2IL-2Rγ-deficient C57BL/6 mice that were adoptively transferred with CD4^+^ or CD8^+^ T cells isolated from *L. sigmodontis*-infected C57BL/6 mice 28 days p.i. were analysed for individual **(A)** female and **(B)** male worm length and **(C)** female and **(D)** male worm length per mouse. In addition, microfilaria (MF) counts were determined in the **(E)** thoracic cavity (TC) and **(F)** peripheral blood (PB) and **(G)** during the course of infection in PB upon 50 days p.i. Moreover, female adult worms were analysed to determine **(H)** embryonic stages. Graphs show dot blots with median and interquartile ranges from individual mice from **(A–G)** three independent experiments including no adoptive transfer (PBS; n=23) and adoptive transfer of CD4^+^ (n=21) and CD8^+^ T cells (n=25) into RAG2IL-2Rγ-deficient C57BL/6 mice. **(H)** Results are from one experiment including n=20, n=4 and n=20 individual worms from no adoptive transferred (PBS; n=7) and CD4^+^ (n=2) and CD8^+^ T cell (n=8) adoptive transferred RAG2IL-2Rγ-deficient C57BL/6 mice, respectively. Significant differences between the groups were determined by Kruskal-Wallis-test followed by a Dunn’s multiple comparison test.

Since adoptive transfer of naïve T cells did not significantly reduce worm burden, we adoptively transferred CD4^+^ or CD8^+^ T cells from C57BL/6 donor mice that were infected with *L. sigmodontis* for 28 days, a time point when adult worms reside in the TC (38). Interestingly, adoptive transfer of CD4^+^ but not CD8^+^ T cells from *L. sigmodontis*-infected C57BL/6 donor mice led to a significant reduction of worm numbers in the TC of *L. sigmodontis*-infected RAG2IL2Rγ-deficient mice at day 72 p.i. in comparison to no (PBS) and CD8^+^ T cell transferred mice (**Figures 4A–C**). Moreover, transfer of CD4^+^ T cells from *L. sigmodontis*-infected donor mice provoke worm encapsulation and nodule formation (**Figures 4D, E**), which ultimately leads to the clearance of adult worms accompanied with reduce inflammation score around the lung and diaphragm tissue (**Figure 4F**). Despite the fact that adult worms were only rarely isolated (8 out of 21 mice harbour adult worms) from RAG2IL2Rγ-deficient mice that received CD4^+^ T cells from *L. sigmodontis*-infected C57BL/6 donor mice, individual worm length as well as worm length per individual mouse was reduced compared to PBS- or CD8^+^ T cell-injected mice (**Figures 5A–D**). The clearance of the adult worms also results in a complete loss of MF at the site of infection and periphery (**Figures 5E, F**). Interestingly, adoptive transfer of CD4^+^ T cells from *L. sigmodontis*-infected donor mice even prevented the onset of MF release in RAG2IL2Rγ-deficient mice, since no MF could be determined upon day 50 p.i. compared to increased MF loads in peripheral blood from PBS- and CD8^+^ T cell-injected mice (**Figure 5G**). Consequently, almost no embryonic stages could be observed in the rarely recovered female worms (**Figure 5H**), showing that transfer of CD4^+^ T cells from C57BL/6 donor mice that have an ongoing *L. sigmodontis* infection drives anti-filarial immune responses that clear and harm the majority of adult worms preventing mating and fertilisation of female worms.

**Figure 6:**
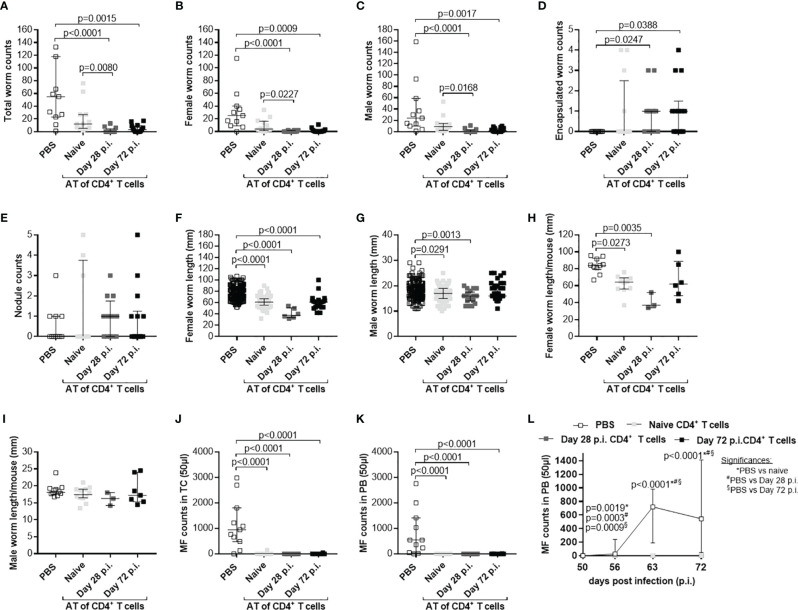
Comparable efficacy of CD4^+^ T cells isolated from day 28 or 72 *L. sigmodontis*-infected C57BL/6 donor mice. CD4^+^ T cells from C57BL/6 donor mice that were either naïve or infected with *L. sigmodontis* for 28 or 72 days were injected intravenously (i.v.) into the tail vein of RAG2IL-2Rγ-deficient C57BL/6 mice one day prior to *L. sigmodontis* infection. On day 72 p.i. mice were analysed to assess **(A)** total, **(B)** female, **(C)** male and **(D)** encapsulated worm counts as well as **(E)** nodule counts in the thoracic cavity (TC). Moreover, individual **(F)** female and **(G)** male worm length and **(H)** female and **(I)** male worm length per mouse were measured. Finally, microfilaria (MF) counts were determined in the **(J)** TC and **(K)** peripheral blood (PB) and **(L)** during the course of infection upon 50 days p.i. in PB. **(A–L)** Graphs show dot blots with median and interquartile ranges from individual mice from two independent experiment including no adoptive transfer (PBS; n=11) and adoptive transfer of CD4^+^ T cell from naïve donor mice (n=12) or donor mice that were infected with *L. sigmodontis* for 28 days (n=12) or 72 days (n=14) into RAG2IL-2Rγ-deficient C57BL/6 mice. Significant differences between the groups were determined by Kruskal-Wallis-test followed by a Dunn’s multiple comparison test. AT, adoptive transfer.

Based on those results, we aim to decipher if the time point of adoptive transfer and the infection status of the adoptively transferred cells influence parasite clearance. Therefore, first we adoptively transferred CD4^+^ T cells from naïve and *L. sigmodontis*-infected (day 28 p.i.) C57BL/6 donor mice into *L. sigmodontis*-infected RAG2IL-2Rγ-deficient C57BL/6 mice on day 28 p.i. (L4 become adult and reside in the TC) and day 49 p.i. (onset of MF; **Supplementary Figure 1B**). Interestingly, only individual worm lengths were significantly reduced upon adoptive transfer of CD4^+^ T cells from *L. sigmodontis*-infected donor mice independently on the time point of adoptive cell transfer, whereas worm and MF counts were comparable in all groups indicating that CD4^+^ T cells cannot clear an established *L. sigmdontis* infection (**Supplementary Figure 8**). Secondly, we decipher if CD4^+^ T cells from donor mice that already cleared the *L. sigmodontis* infection can efficiently clear parasite burden. Thus, we adoptively transferred CD4^+^ T cells from donor mice that were *L. sigmodontis*-infected for 72 days, a time point when C57BL/6 mice already cleared the infection, into RAG2IL-2Rγ-deficient C57BL/6 mice one day prior *L. sigmodontis* infection (**Supplementary Figure 1A**). Interestingly, CD4^+^ T cells from day 72 *L. sigmodontis-*infected donor mice efficiently reduced the worm burden and MF loads comparable to CD4^+^ T cells from day 28 *L. sigmodontis*-infected C57BL/6 donor mice (**Figure 6**), suggesting that an ongoing *L. sigmodontis* infection primes CD4^+^ T cells leading to a long-term memory against this filaria. However, there was a tendency that CD4^+^ T cells from donor mice that already cleared the *L. sigmodontis* infection (day 72 p.i.) were not able to reduce efficiently the length of the remaining male worms (**Figures 6G, H**).

In summary, these results demonstrate that adoptive transfer of CD4^+^ T cells from C57BL/6 donor mice with an ongoing (day 28 p.i.) or cleared (day 72 p.i.) *L. sigmodontis* infection effectively reduces worm and MF burden in *L. sigmodontis*-infected RAG2IL-2Rγ-deficient C57BL/6 mice. However, this partial protection strongly depends on the time point of adoptive transfer, since CD4^+^ T cell transfer could not reduce parasite numbers when *L. sigmodontis* worms are already reside in the TC.

### Immune Profile of the Adoptively Transferred CD4^+^ T Cells and Host Responses in *L. sigmodontis*-Infected RAG2IL-2Rγ-Deficient Mice

**Figure 7:**
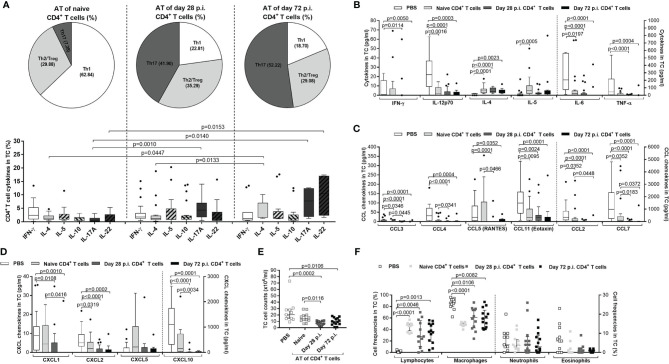
Immune profile of transferred CD4^+^ T cells in *L. sigmodontis*-infected RAG2IL-2Rγ^-/-^ mice. On day 72 p.i., CD4^+^ T cells and immune responses in the thoracic (TC) were analysed from *L. sigmodontis*-infected RAG2IL-2Rγ-deficient C57BL/6 mice. Therefore, CD4^+^ T cells that were either adoptively transferred from naïve, or *L. sigmodontis-*infected (day28 or day 72 p.i.) C57BL/6 donor mice (day28 or day 72 p.i.) were isolated and analysed for cytokine secretion patterns in the **(A)** TC using flow cytometry. Moreover, **(B)** cytokine and **(C, D)** chemokine levels were also analysed in the TC. Finally, **(E)** total TC cell counts and **(F)** frequencies of lymphocytes, macrophages, neutrophils and eosinophils were analysed using cytospin technique. Graphs show **(A)** pie charts, **(A–D)** box and whiskers and **(E, F)** dot blots with median and interquartile ranges. Graphs show **(A)** four independent experiments including adoptive transfer of CD4^+^ T cells from naïve donor mice (n= 24) or donor mice that were infected with *L. sigmodontis* for 28 days (n=21) or 72 days (n=13), **(B–D)** 6 independent experiments including no adoptive transfer (PBS; n=25) and adoptive transfer of CD4^+^ T cells from naïve donor mice (n= 22) or donor mice that were infected with *L. sigmodontis* for 28 days (n=21) or 72 days and **(E, F)** 2 independent experiments including PBS (n=11) and adoptive transfer of CD4^+^ T cells from naïve donor mice (n= 12) or donor mice that were infected with *L. sigmodontis* for 28 days (n=12) or 72 days (n=14) into RAG2IL-2Rγ-deficient C57BL/6 mice. Significant differences between the groups were determined by Kruskal-Wallis-test followed by a Dunn’s multiple comparison test. AT, adoptive transfer.

Since CD4^+^ T cells from *L. sigmodontis*-infected C57BL/6 donor mice showed a higher efficacy to reduce worm and MF burden compared to CD4^+^ T cells from naïve donor mice, we characterised cytokine patterns of the adoptively transferred CD4^+^ T cells from RAG2IL-2Rγ-deficient C57BL/6 mice on day 72 p.i. at the site of infection (TC). In detail, TC cells were isolated and frequencies of IFN-γ+ (Th1), IL-4+, IL-5+ and IL-10+ (Th2/regulatory) and IL-17A+ and IL-22+ (Th17) CD4^+^ T cells assessed using flow cytometry. Although, CD4^+^ T cell frequencies were comparable in the TC of RAG2IL-2Rγ-deficient mice that received CD4^+^ T cells from naive or *L. sigmodontis*-infected C57BL/6 donor mice, CD4^+^ T cells that were adoptively transferred from day 28 or day 72 *L. sigmodontis*-infected C57BL/6 donor mice revealed increased Th17 immune responses compared to CD4^+^ T cells that were obtained from naïve C57BL/6 donor mice (**Figure 7A**). Moreover, detailed comparison of the cytokine secretion patterns showed that RAG2IL-2Rγ-deficient C57BL/6 mice that were adoptively transferred with CD4^+^ T cells from day 28 and day 72 *L. sigmodontis*-infected C57BL/6 donor mice had higher frequencies of CD4^+^IL-17A^+^T cells. In addition, CD4^+^ T cells that were derived from day 72 *L. sigmodontis*-infected donor mice and adoptively transferred into RAG2IL-2Rγ-deficient C57BL/6 mice showed increased expression of IL-22 and IL-4 compared to adoptively transferred CD4^+^ T cells from naïve or day 28 *L. sigmodontis*-infected C57BL/6 donor mice, respectively (**Figure 7A**). These results suggest that the efficient clearance of parasite burden depends on the polarization of donor CD4^+^ T cells into a Th17 phenotype upon transfer into recipients. However, since adoptively transferred CD4^+^ T cells showed distinct cytokine patterns in RAG2IL-2Rγ-deficient mice upon 72 days p.i. (**Figure 7A**), we next analysed the overall cytokines and chemokines levels in the TC using a 36 cytokine and chemokine luminex kit. From the 36 analytes, 10 (IL-1β, IL-10, IL-13, IL-15, IL-17A, IL-22, IL-27, G-SCF, GM-CSF and LIF) were below the detection limit. However, IFN-γ, IL-12p70, IL-6 and TNF-α were significantly reduced in RAG2IL-2Rγ-deficient mice that received CD4^+^ T cell, especially from *L. sigmodontis*-infected donors (**Figure 7B**). Interestingly, only the Th2 associated cytokines IL-4 and IL-5 were increased in CD4^+^ T cells transferred into RAG2IL-2Rγ-deficient recipients on day 72 p.i. and (**Figure 7B**), suggesting that Th2 associated cytokines play a crucial role for parasite clearance despite the fact that transferred CD4^+^ T cells did not show a prominent Th2 phenotype (**Figure 7A**). However, chemokines like CCL2, CCL3, CCL4, CCL5, CCL7 and CCL11 (**Figure 7C**) and CXCL1, CXCL2, CXCL5 and CXCL10 (**Figure 7D**) were again reduced in RAG2IL-2Rγ-deficient mice that received CD4^+^ T cells, especially from *L. sigmodontis*-infected donors. The overall reduction of cytokines and chemokines in the TC especially in mice that received CD4^+^ T cells from *L. sigmodontis*-infected donors confirms the previous findings that adult worms were eliminated in the TC of the RAG2IL-2Rγ-deficient mice (**Figures 5**, **6**) leading also to reduced infiltration and accumulation of immune cells at the site of infection on day 72 p.i. Indeed, the overall cell numbers in the TC were reduced in RAG2IL-2Rγ-deficient mice that were transferred with CD4^+^ T cells derived from *L. sigmodontis*-infected C57BL/6 donor mice (**Figure 7E**). Finally, we assessed the composition of immune cells in the TC using the cytospin technique and revealed as expected that no lymphocytes were present at the site of infection in mice that did not receive adoptive transfer of CD4^+^ T cells (PBS control), whereas CD4^+^ T cell transferred mice showed significantly increased numbers of lymphocytes (**Figure 7F**). Interestingly, the lack of lymphocytes in the PBS control group was compensated by increased macrophage frequencies compared to CD4^+^ T cell transferred mice, whereas neutrophil and eosinophil frequencies were comparable between the groups (**Figure 7F**). In general, no differences in cell frequencies within the CD4^+^ T cell transferred mice groups could be observed, suggesting that the clearance of parasite burden depends on infectious status and function (Th17 polarization) of the CD4^+^ T cells rather than cell number and presence of innate immune cells highlighting the central role of CD4^+^ T cells for immunity against *L. sigmodontis*.

## Discussion

The worldwide distribution and high infection rates in tropical areas highlights the successful survival strategy and adaptability of filarial nematodes. Filariae developed complex life cycles in which insects (vectors) and humans are used as host for transmission and reproduction, respectively. Although millions of humans are infected and suffer from severe pathology, knowledge about parasite evasion and survival tactics are not fully understood. Over the last decades, several studies showed that filariae modulate immunity in humans through the induction of Th2 and regulatory T and B cell populations accompanied with the suppression of pro-inflammatory and Th1 immune responses to guarantee long-term survival and reproduction of the parasite (10–19, 63). Nevertheless, it is impossible to investigate the complexity of the filarial-driven modulation and survival tactics solely in humans due to limited access to parasitic life stages, unknown infection time point, possible re- and co-infections and most importantly ethical concerns. Although *in vitro* culture models of human filariae are promising (25–30) and experimental infections in monkeys have been established (64, 65), these approaches do not reflect the complexity of the human host and thus cannot be used to study filarial-driven modulation of host immunity and are difficult to perform accompanied with ethical concerns, respectively. Therefore, the rodent model of filariasis, namely *L. sigmodontis*, is used for filarial research and expanded the knowledge about filarial-driven immune regulation and is also a suitable platform for anti-filarial drug testing (36, 37). Mice strains on BALB/c background are susceptible to *L. sigmodontis* whereas others clear the infections before worms get fertile and produce the offspring (38–40). Thus, researchers used knockout BALB/c strains to perform human filarial infections but until know the complete life cycle could not be obtained in rodents (31–35). However, these studies showed that the knockout of the RAG2 gene in BALB/c mice, which result in an impaired maturation of T and B cells, might be a suitable model for human filarial infections. Indeed, this knockout strain was recently used to obtain *Loa loa* larvae stages and adult worms (34, 35) that were used to investigate *Loa loa*-specific immune responses (66). In addition, we showed that the combined knockout of the RAG2 gene and IL-2Rγ chain, which leads to a complete loss of T, B and natural killer cells (55), in semi-susceptible C57BL/6 mice results into 100% patency, higher worm numbers (60) and long-lasting secretion of MF, in contrast to susceptible knockout BALB/c mice (46, 47).

To elucidate the potential of the RAG2IL-2Rγ-deficient mouse model, we here established adoptive transfer experiments which can be used for in-depth analysis of immunity against filariae. We revealed that adoptive transfer of CD4^+^, but not CD8^+^ T cells obtained from *L. sigmodontis*-infected C57BL/6 donor mice one day prior to *L. sigmodontis* infection of RAG2IL-2Rγ-deficient mice significantly reduces parasite burden. Interestingly, adoptive transfer during an established *L. sigmodontis* infection, when adult worms are present (day 28 p.i.) or shortly before the onset of MF release (day 49 p.i.), did not reduce parasite burden, suggesting that CD4^+^ T cells play a crucial role especially for the protection against filarial infection and impair the development of larval stages. Until now the role of CD4^+^ T cells during *L. sigmodontis* infection was only elucidated in BALB/c mice (41) and thus this study showed to our knowledge the first time the crucial role of CD4^+^ T cells for anti-filarial immunity in C57BL/6 mice. Since information about the role of adaptive immune cells for anti-filarial immunity in C57BL/6 mice is missing, further experiments should elucidate also the role of B cells by using the established adoptive cell transfer model in RAG2IL-2Rγ-deficient mice, especially since B cells have been shown to be important for the immunity in BALB/c strains (42) and distinct immunoglobulin patterns have been shown to be induced during human filarial infection that are crucial for the regulation of host immunity (11, 67–69).

In addition, the findings from the CD4^+^ T cells transfers also revealed that a previous *L. sigmodontis* infection influences an efficient anti-filarial response, since CD4^+^ T cells from naïve C57BL/6 donor mice did reduced MF numbers and worm length but not adult worm burden, whereas CD4^+^ T cells isolated from *L. sigmodontis*-infected C57BL/6 donor mice, which harbour adult worms in the TC (day 28 p.i.) led to drastically reduced worm and MF burden as well as worm length accompanied with significantly reduced inflammation around lung and diaphragm tissue. Interestingly, similar results were also obtained by CD4^+^ T cells that were isolated from C57BL/6 donor mice that already naturally cleared the infection (day 72 p.i.). These findings highlight that CD4^+^ T cells can develop a memory towards *L. sigmodontis* that enables efficient clearance of the parasites. Indeed, it have been shown that during chronic lymphatic filariasis (70) and *Brugia pahangi* infection (71) T cell memory is initiated and that primed T cells are able to clear the infection efficiently. Further adoptive transfer experiments should concentrate on distinct T cells subsets like effector, memory or effector memory CD4^+^ T cells in combination with B cells to elucidate distinct mechanisms of the anti-filarial immunity. Furthermore, although lack of IL-4 and IL-10 by transferred T cells did not seem to influence the ability of naïve CD4^+^ T cells to reduce worm length and MF numbers, the role of those cytokines should be investigated further *via* the adoptive transfer of CD4^+^ T cells from *L. sigmodontis*-infected IL-4, IL-10 or IL-5-deficient mice, since Th2 and regulatory cytokines were shown to influence anti-filarial immunity in C57BL/6 and BALB/c mice (44–47) as well as during human filarial infections (10, 12, 13, 16, 17, 19, 63, 72). Moreover, on day 72 p.i., IL-4 and IL-5 were the only cytokines which were increased in the TC of recipient mice following a transfer of CD4^+^ T cells, whereas the majority of the cytokines and chemokines were actually reduced at the site of infection. The increased Th2 cytokines might stem from innate immune cells like antigen-presenting cells or granulocytes because the adoptively transferred CD4^+^ T cells from naïve and *L. sigmodontis*-infected donor mice are mainly characterized by a Th1 and Th17 profile, respectively. Since, CD4^+^ T cells from *L. sigmodontis*-infected donor mice clear efficiently worm burden and showed increased Th17 immune responses compared to CD4^+^ T cells that were obtained from naïve C57BL/6 donor mice, we can conclude that Th17 polarization may play an essential role for anti-filarial immunity. Indeed, the important role of Th17 immune responses during anti-filarial immunity has been previously reported in mice and humans (14, 54, 73). Nevertheless, further experiments, including adoptive transfers of IL-17-deficient CD4^+^ T cells and analysis of earlier time points, such as day 28 p.i. (development of adult worms) and day 49 p.i. (the onset of MF), should be performed to decipher the functional role of Th17 cells and the development of host immune responses against different life stages *in vivo*, respectively. In addition, to investigate possible reasons why and how CD4^+^ T cells derived from *L. sigmodontis*–infected donor mice efficiently clear parasite burden compared to cells from naïve donor mice, immune profiling of splenic CD4^+^ T cells prior to adoptive transfer needs to be performed. However, detection of cytokine expression levels by splenic CD4^+^ T cells was inconclusive due to low cytokine expression levels. Therefore, *in vitro* re-stimulation experiments with filarial antigens need to be performed in future studies to assess the immune profile of splenic CD4^+^ T cell prior adoptive transfer.

In conclusion, this study suggests for the first time that CD4^+^ T cells are central for the immunity against *L. sigmodontis* in C57BL/6 mice and that clearance of the parasite depends on infectious status and Th17 polarization of the CD4^+^ T cells. In general, adoptive transfer experiments using RAG2IL-2Rγ-deficient C57BL/6 mice provide an optimal platform to decipher cell subsets and their corresponding immune responses that are crucial for anti-filarial immunity. Since our recent studies already showed that RAG2IL-2Rγ-deficient C57BL/6 mice are susceptible to the human filaria *Loa loa* (35, 66, 74), this platform is not restricted to rodent filariae but will also be a suitable tool for in-depth research on human filariae and preclinical research, especially might allow the identification of essential immunological component(s) and accurate predictions of the efficacy of macrofilaricidal drugs, like flubendazole (75), oxfendazole (76) or emodepside (77).

## Data Availability Statement

The original contributions presented in the study are included in the article/**Supplementary Material**. Further inquiries can be directed to the corresponding author.

## Ethics Statement

The animal study was reviewed and approved by German animal protection laws and EU guidelines 2010/63/E4 Landesamt für Natur, Umwelt und Verbraucherschutz Nordrhein-Westfalen, Recklinghausen, Germany (84.02.04.2017.A122).

## Author Contributions

MR, SW, and LEL conceived and designed the study. MR, AW, KA, LMW, RSET, DB-W, VCC, and CAK performed and analysed the experiments. MR drafted the manuscript while AW, KA, LMW, RSET, AH, and SW critically revised the article and controlled the intellectual content. All authors contributed to the article and approved the submitted version.

## Funding

This work was supported by the German Research Foundation (DFG) within the German-African Projects in Infectiology (MARAG project) [RI 3036/1-1 to MR, LEL and SW] and in addition, MR is financially supported by the German Federal Ministry of Education and Research (BMBF) and the Ministry of Culture and Science of the State of North Rhine-Westphalia (MKW) within the framework of the Excellence Strategy of the Federal and State Governments. RSET received a scholarship awarded by the German Academic Exchange Committee (DAAD) and her current position is funded by the DFG [LA2746/5]. Furthermore, AH and SW are also supported by the BMBF [01KA1611 and 01KA2027] and AH is additionally funded by the Deutsche Forschungsgemeinschaft (DFG, German Research Foundation) under Germany’s Excellence Strategy – EXC2151 – 390873048 and as a member of the German Centre of Infectious Disease (DZIF).

## Conflict of Interest

The authors declare that the research was conducted in the absence of any commercial or financial relationships that could be construed as a potential conflict of interest.

## Publisher’s Note

All claims expressed in this article are solely those of the authors and do not necessarily represent those of their affiliated organizations, or those of the publisher, the editors and the reviewers. Any product that may be evaluated in this article, or claim that may be made by its manufacturer, is not guaranteed or endorsed by the publisher.
