# Supplementary material for: Adoptive Transfer of Immune Cells Into RAG2IL-2Rγ-Deficient Mice During Litomosoides sigmodontis Infection: A Novel Approach to Investigate Filarial-Specific Immune Responses
Source: Front Immunol. 2021 Nov 18;12:777860. doi: 10.3389/fimmu.2021.777860 (PMC8636703; doi:10.3389/fimmu.2021.777860)
Supplement: Supplementary file 1 [file DataSheet_1.docx]

Supplementary Material

**
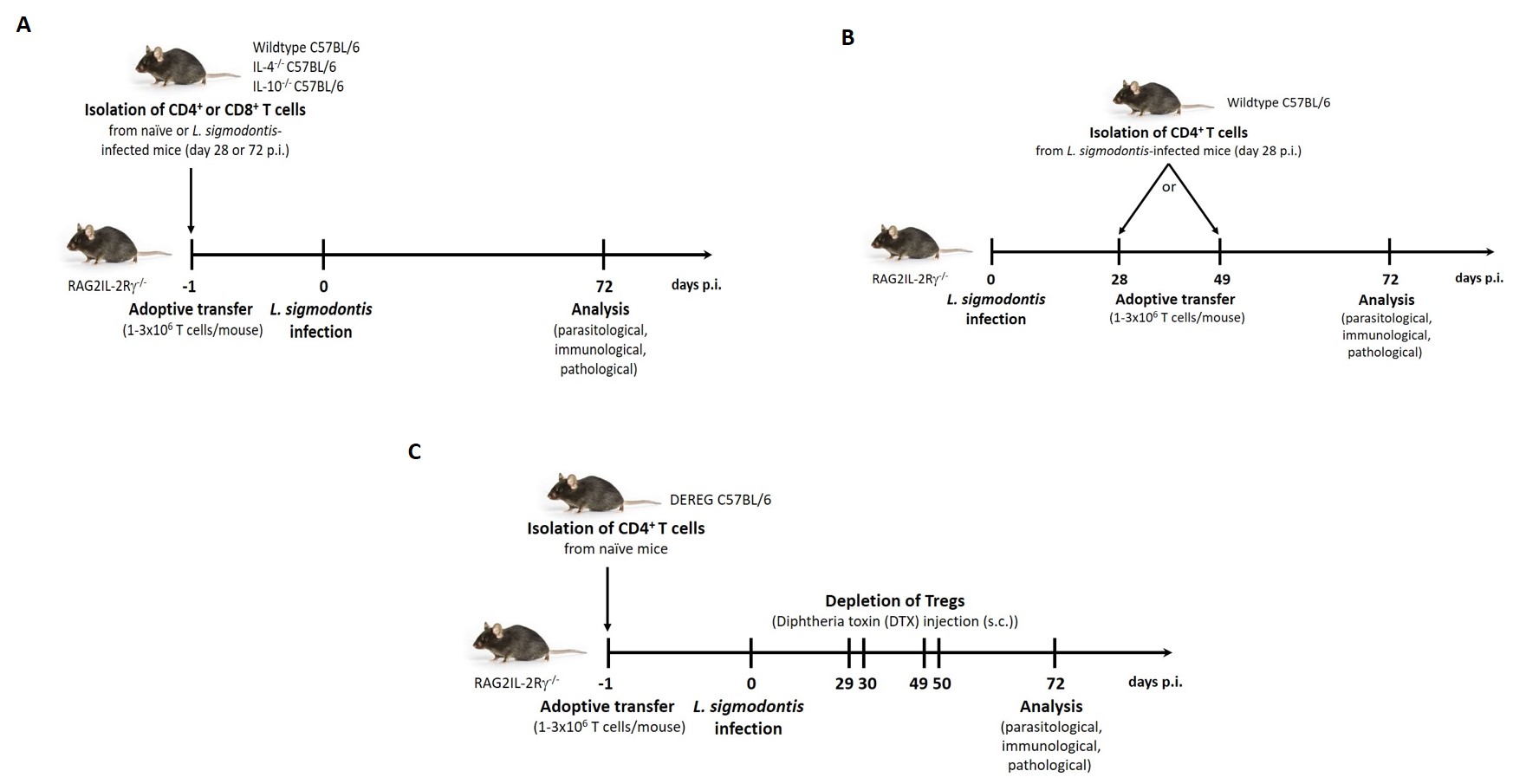
**

**Supplementary Figure 1: Scheme of experimental layouts of *L. sigmodontis* infection and T cell adoptive transfer experiments using RAG2IL-2Rγ^-/-^ mice**

Mice were naturally infected with *L. sigmodontis* and CD4^+^ or CD8^+^ T cells from naïve and 28- or 72-days *L. sigmodontis*-infected wildtype, IL-4^-/-^ or IL-10^-/-^C57BL/6 donor mice were injected intravenously (i.v.) into the tail vain of RAG2IL-2Rγ-deficient C57BL/6 mice **(A)** one day prior to *L. sigmodontis* infection or **(B)** 28 or 49 days p.i.. **(C)** Moreover, upon adoptive transfer of T cells from DEREG C57BL/6 donor mice regulatory T cells were depleted through i.p. injection of diphtheria toxin on day 29 and 30 and day 49 and 50 p.i..

**
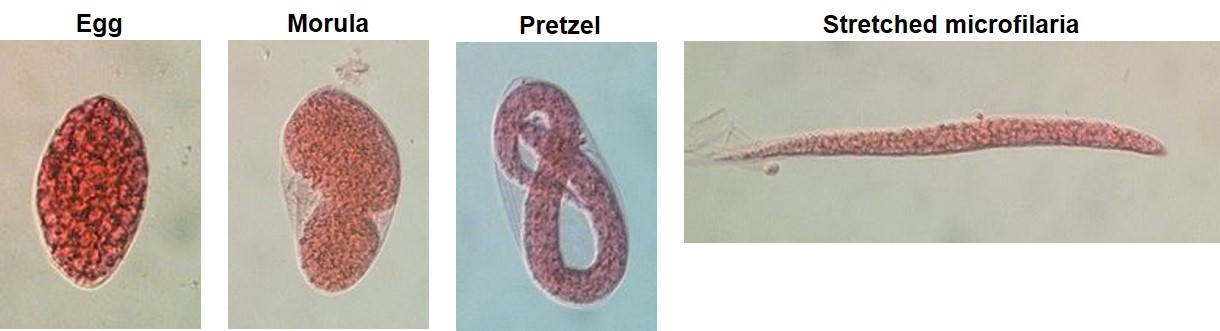
**

**Supplementary Figure 2: Embryogenic stages of *L. sigmodontis* in female adult worms**

Microscopical pictures of embryonic egg, morula, pretzel and stretched microfilaria stages of *L. sigmodontis* female adult worms.


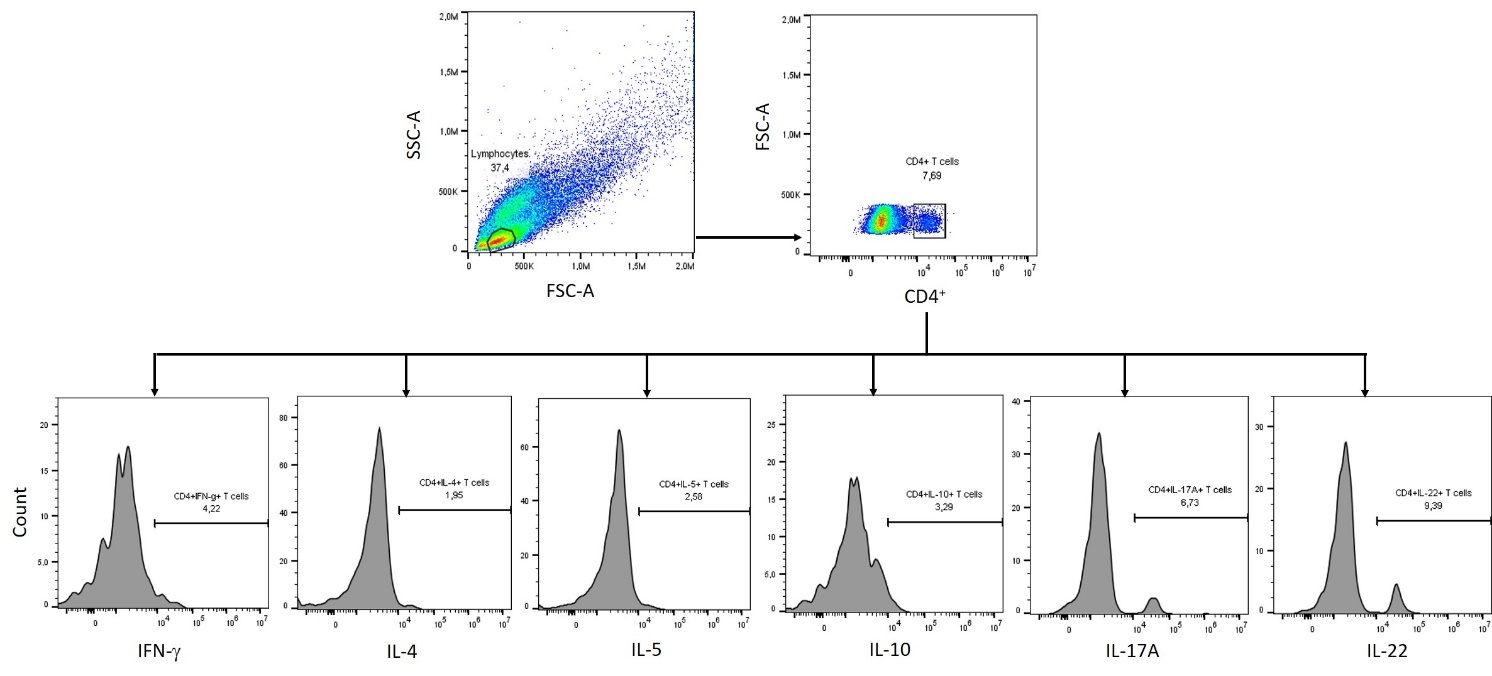


**Supplementary Figure 3: Gating strategy of CD4^+^ T cells and their cytokine expression**

Thoracic cavity fluid of RAG2IL-2Rγ-deficient mice that were transferred with CD4^+^ T cell and infected for 72 days with *L. sigmodontis* were investigated for CD4^+^ T cells and their cytokine production. Therefore, lymphocytes were gated using the forward scatter-area (FSC-A) and side scatter -area (SSC-A) before separated to CD4^+^ T cells by gating CD4 expression. Finally, frequencies of cytokine expression (IFN-γ, IL-4, IL-5, IL-10, IL-17A and IL-22) of the CD4^+^ T cells were analysed using a histogram plot. Fluorescence minus one (FMO) controls were acquired to discriminate populations.

**
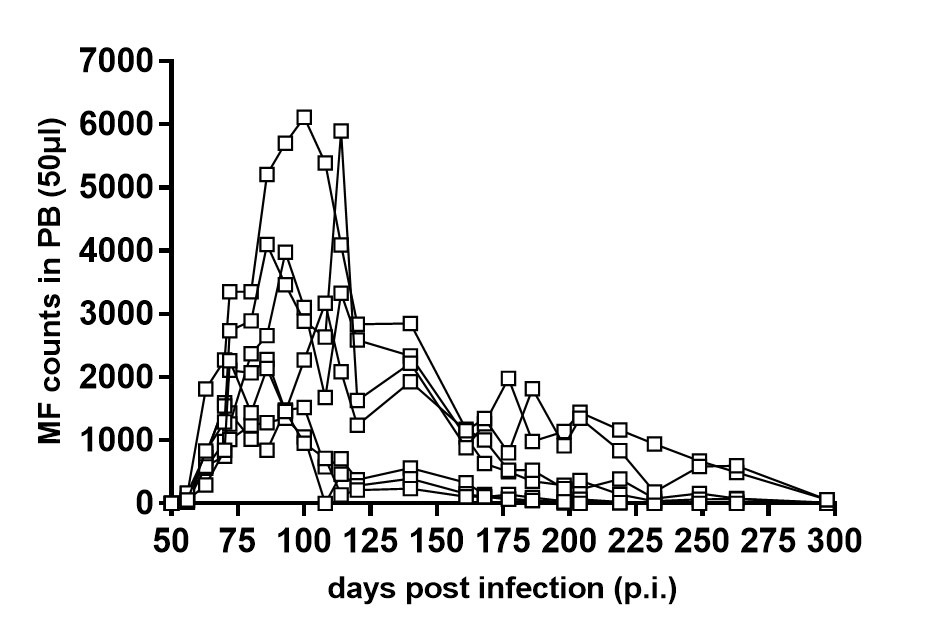
**

**Supplementary Figure 4: Long-term patency of RAG2IL-2Rγ-deficient mice**

RAG2IL-2Rγ-deficient mice were naturally infected with *L. sigmodontis* and release of microfilariae (MF) in peripheral blood (PB) were regularly measured during an ongoing infection. Results are shown from one infection experiment including 7 mice.


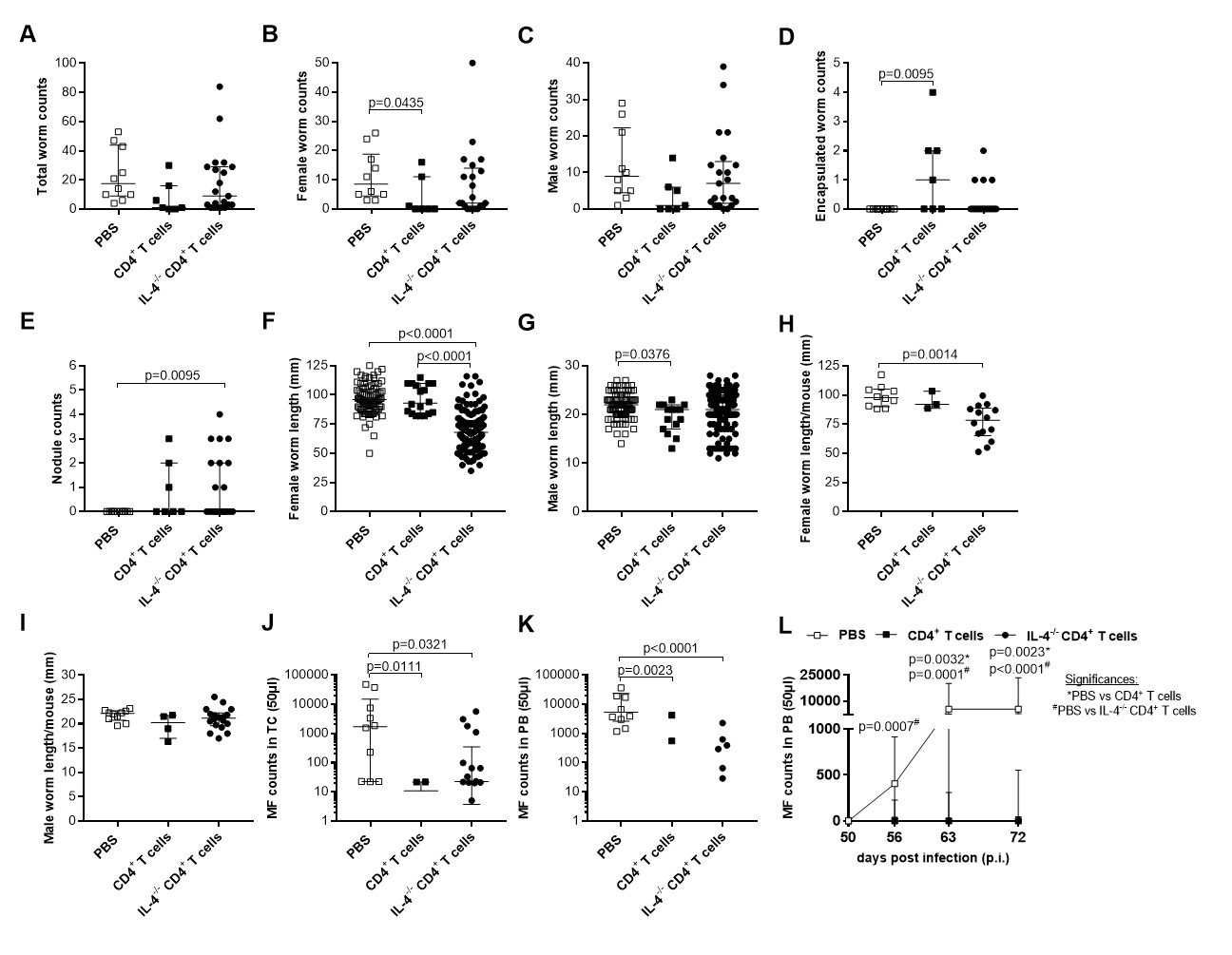


**Supplementary Figure 5: Role of IL-4 during adoptive transfer of CD4^+^ T cells from naïve C57BL/6 donor mice**

CD4^+^ T cells from naïve wildtype and IL-4^-/-^ C57BL/6 donor mice were injected intravenously (i.v.) into the tail vain of RAG2IL-2Rγ-deficient C57BL/6 mice one day prior to *L. sigmodontis* infection. On day 72 p.i. mice were analysed to assess **(A)** total, **(B)** female, **(C)** male and **(D)** encapsulated worm counts as well as **(E)** nodule counts in the thoracic cavity. Moreover, individual **(F)** female and **(G)** male worm length and **(H)** female and **(I)** male worm length per mouse were measured. Finally, microfilaria (MF) counts were determined in the **(J)** thoracic cavity (TC) and **(K)** peripheral blood (PB) on day 72 p.i.. and **(L)** during the course of infection upon 50 days p.i. in PB. **(A-L)** Graphs show dot blots with median and interquartile ranges from three independent experiments including no adoptive transfer (PBS; n=10) and adoptive transfer of CD4^+^ (n=7) and IL-4^-/-^ CD4^+^ T cells (n=21) into RAG2IL-2Rγ-deficient C57BL/6 mice. Significant differences between the groups were determined by Kruskal-Wallis-test followed by a Dunn’s multiple comparison test.


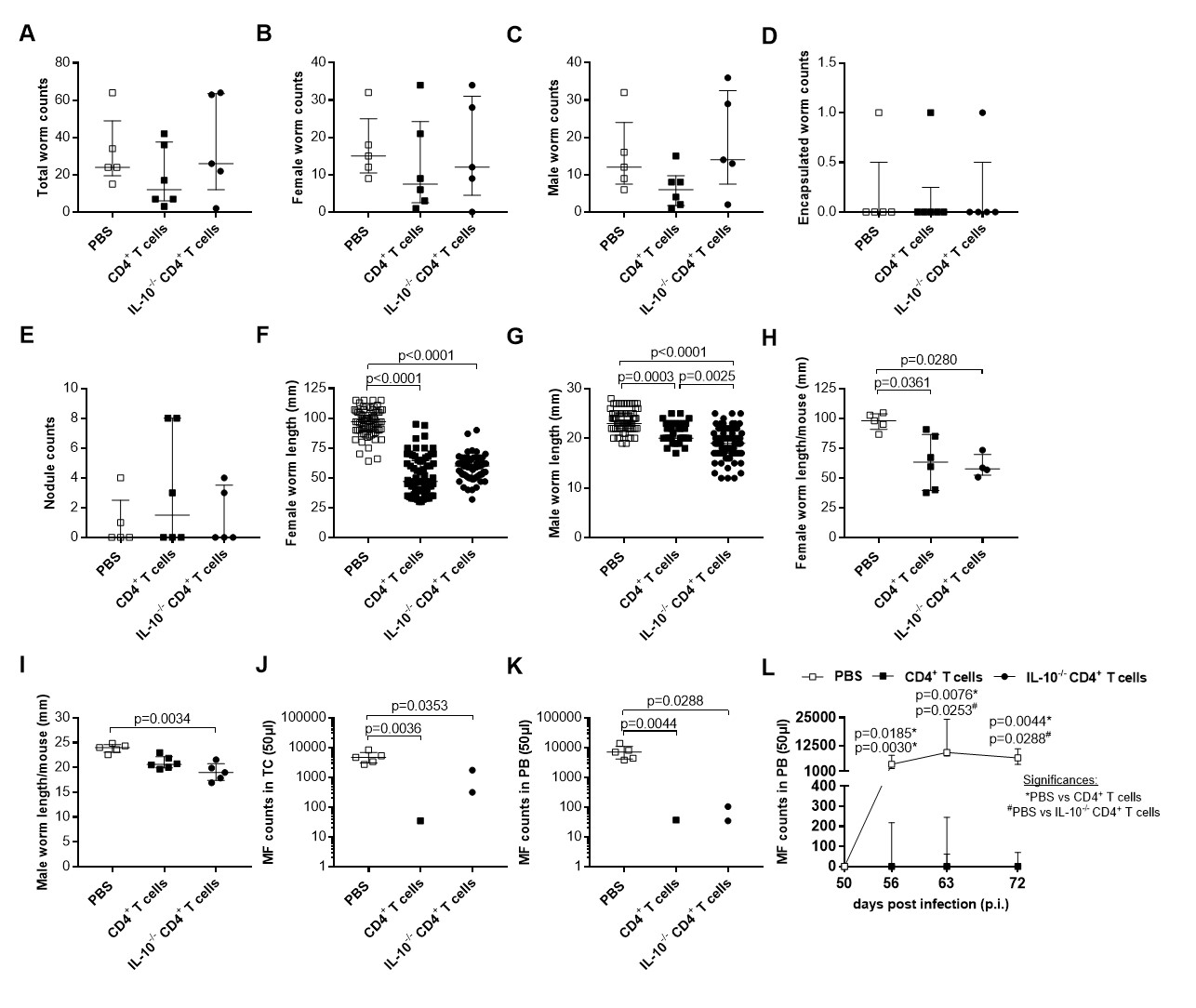


**Supplementary Figure 6: Role of IL-10 during adoptive transfer of CD4^+^ T cells from naïve C57BL/6 donor mice**

CD4^+^ T cells from naïve wildtype and IL-10^-/-^ C57BL/6 donor mice were injected intravenously (i.v.) into the tail vain of RAG2IL-2Rγ-deficient C57BL/6 mice one day prior to *L. sigmodontis* infection. On day 72 p.i. mice were analysed to assess **(A)** total, **(B)** female, **(C)** male and **(D)** encapsulated worm counts as well as **(E)** nodule numbers in the thoracic cavity. Moreover, individual **(F)** female and **(G)** male worm length and **(H)** female and **(I)** male worm length per mouse were measured. Finally, microfilaria (MF) counts were determined in the **(J)** thoracic cavity (TC) and **(K)** peripheral blood (PB) on day 72 p.i. and **(L)** during the course of infection upon 50 days p.i. in PB. **(A-L)** Graphs show dot blots with median and interquartile ranges from one experiment including no adoptive transfer (PBS; n=5) and adoptive transfer of CD4^+^ (n=6) and IL-10^-/-^ CD4^+^ T cells (n=5) into RAG2IL-2Rγ-deficient C57BL/6 mice. Significant differences between the groups were determined by Kruskal-Wallis-test followed by a Dunn’s multiple comparison test.


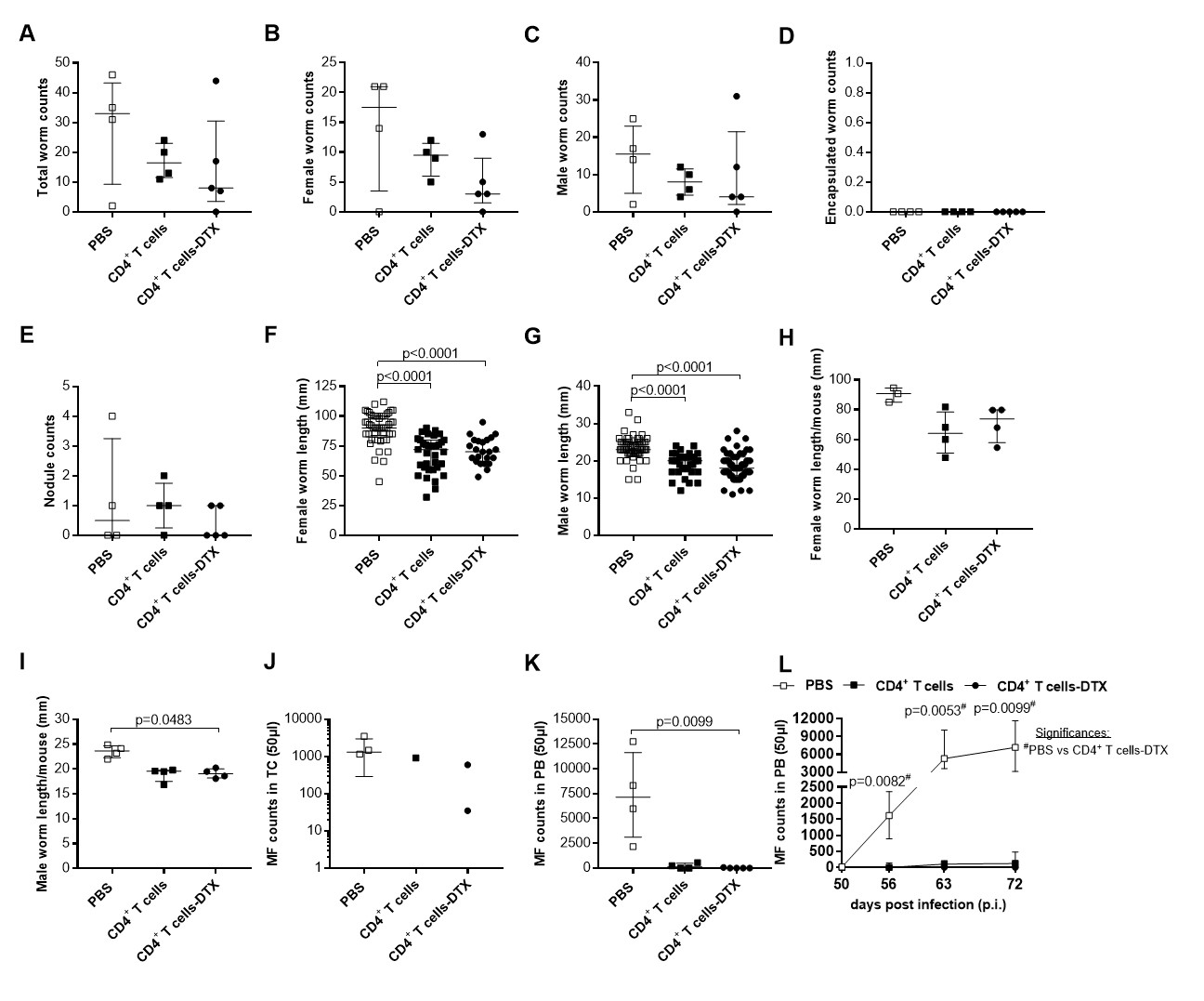


**Supplementary Figure 7: Role of regulatory T cells during adoptive transfer of CD4^+^ T cells from naïve C57BL/6 donor mice**

CD4^+^ T cells from naïve DEREG C57BL/6 donor mice were injected intravenously (i.v.) into the tail vain of RAG2IL-2Rγ-deficient C57BL/6 mice one day prior to *L. sigmodontis* infection. To deplete regulatory T cells (Tregs) diphtheria toxin (DTX) was intraperitoneal (i.p.) injected into adoptively transferred and *L. sigmodontis*-infected RAG2IL-2Rγ-deficient C57BL/6 mice on day 29 and 30 and day 49 and 50 p.i.. On day 72 p.i. mice were analysed to assess **(A)** total, **(B)** female, **(C)** male and **(D)** encapsulated worm counts as well as **(E)** nodule numbers in the thoracic cavity (TC). Moreover, individual **(F)** female and **(G)** male worm length and **(H)** female and **(I)** male worm length per mouse were measured. Finally, microfilaria (MF) counts were determined in the **(J)** TC and **(K)** peripheral blood (PB) on day 72 p.i.. and **(L)** during the course of infection upon 50 days p.i. in PB. **(A-L)** Graphs show dot blots with median and interquartile ranges from one experiment including no adoptive transfer (PBS; n=4) and adoptive transfer of CD4^+^ (n=4) and CD4^+^ T cells-DTX (Treg depleted; n=5) into RAG2IL-2Rγ-deficient C57BL/6 mice. Significant differences between the groups were determined by Kruskal-Wallis-test followed by a Dunn’s multiple comparison test.


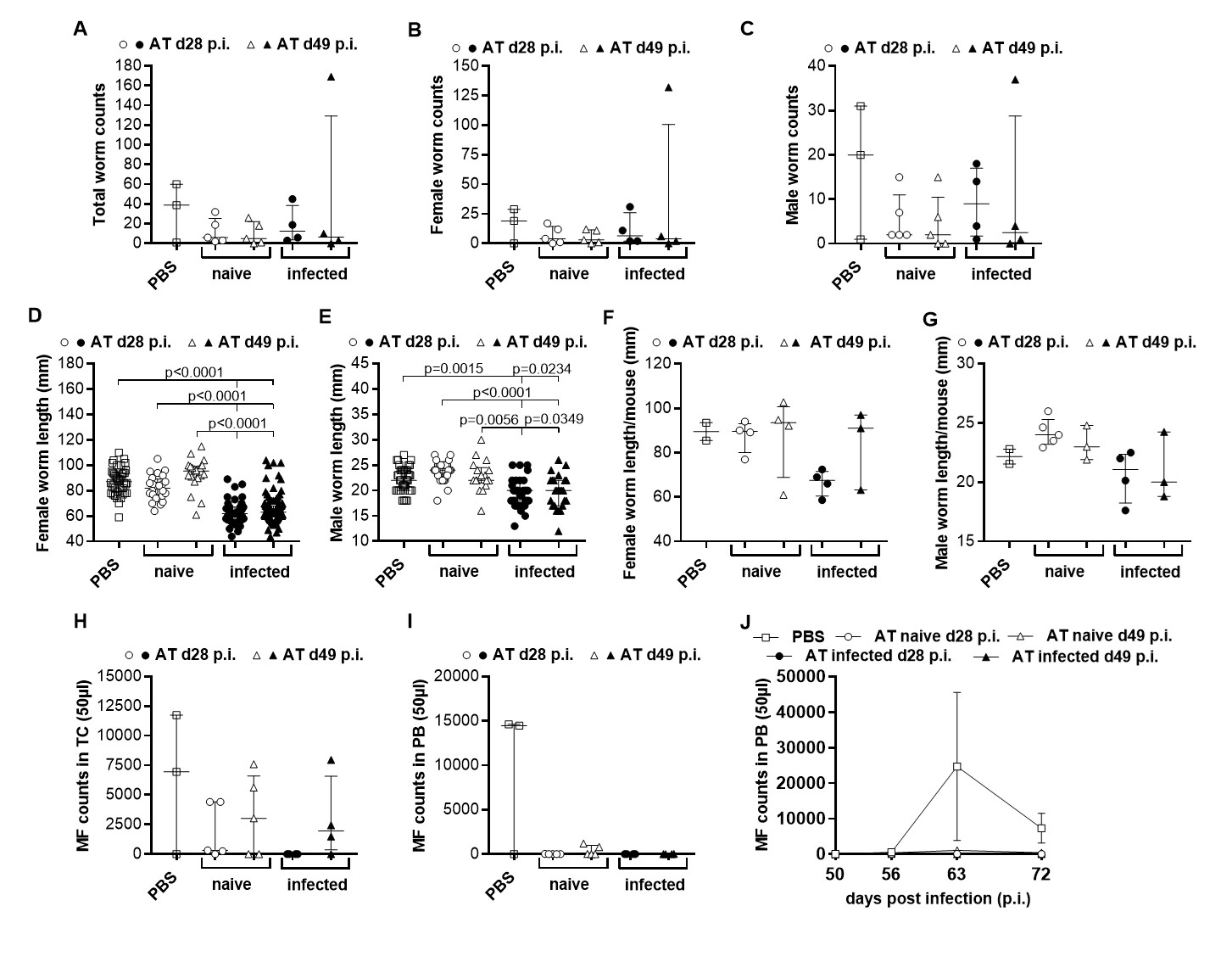


**Supplementary Figure 8: Efficacy of CD4^+^ T cell depends on the time point of the adoptive transfer**

CD4^+^ T cells from C57BL/6 donor mice that were either naïve or infected with *L. sigmodontis* for 28 days were injected intravenously (i.v.) into the tail vain of *L. sigmodontis*-infected RAG2IL-2Rg-deficient C57BL/6 mice on day 28 (AT d29p.i.) or 49 p.i. (AT d49 p.i.). On day 72 p.i. mice were analysed to assess **(A)** total, **(B)** female and **(C)** male worm counts in the thoracic cavity (TC). Moreover, individual **(D)** female and **(E)** male worm length and **(F)** female and **(G)** male worm length per mouse were measured. Finally, microfilaria (MF) counts were determined in the **(H)** TC and **(I)** peripheral blood (PB) and **(J)** during the course of infection upon 50 days p.i. in PB. **(A-J)** Graphs show dot blots with median and interquartile ranges from one experiment including no adoptive transfer (PBS; n=3) and adoptive transfer of CD4^+^ T cells from naïve (naïve) or *L. sigmodontis*-infected (infected) donor mice on day 28 (n=5) or day 49 p.i. (n=4) into RAG2IL-2Rγ-deficient C57BL/6 mice. Significant differences between the groups were determined by Kruskal-Wallis-test followed by a Dunn’s multiple comparison test.
